# Food banking and emergency food aid: expanding the definition of local food environments and systems

**DOI:** 10.1186/s12966-018-0765-2

**Published:** 2019-01-07

**Authors:** Claire Thompson, Dianna Smith, Steven Cummins

**Affiliations:** 1 Department of Public Health, Environment and Society, London School of Hygiene and Tropical Medicine, 15-17 Tavistock Place, London, WC1H 9SH UK; 2 Department of Geography and Environment, University of Southampton, University Road, Southampton, SO17 1BJ UK

**Keywords:** Food insecurity, Food environments, Food aid outlets

## Abstract

If current trends in food insecurity continue then the diets of low-income people may become characterised by the inclusion of significant amounts of donated and surplus food accessed via the third-sector. These developments have yet to be integrated into macro models and concepts of the food environment. Addressing this caveat is necessary in order to both help build an evidence base to challenge policies that exacerbate the drivers of food insecurity and to inform interventions aimed at improving the diets of disadvantaged populations.

Food environments are the collective physical, economic, policy and socio-cultural conditions that influence food and beverage choices and nutritional status [1]. In terms of conceptualising pathways to diet-led health inequalities, landscape metaphors of food deserts, food swamps and food brownfields provide a critical lens for pathologising food environments and systems in low-income and food insecure neighbourhoods [2]. ‘Unhealthy’ food environments are increasingly understood as symptomatic of the interacting pathologies of household poverty, community disadvantage, and the actions of the food industry. Thus, household food insecurity can be understood as an outcome of interrelated social, commercial and economic conditions [3]. While, in this respect, food environments research offers a theoretical framing of food insecurity, it has not, to date, examined the food aid resources and outlets that address food insecurity at the neighbourhood level.

Largely, this is because food banks, and emergency food aid outlets more generally, are charity and third sector run - as opposed to *commercial* retail outlets – meaning that they have tended to fall outside the remit of current food environment research. However, as the food aid sector grows and diversifies, the distinction between not-for-profit and for-profit retail is blurring with subsidised food retail in the form of social enterprises – businesses that reinvest revenue into their organisation and/or the community to tackle social problems [4]. Popular not-for-profit retail formats include community-run shops and social supermarkets that make surplus and donated food available cheaply to those on very low incomes. There is also increasing crossover between food aid and for-profit food retail in the form of corporate social responsibility practices such as regular donations from large non-food companies and commercial supermarkets sponsoring and supporting the set-up of social supermarkets and acting as donation points for food banks.

These developments have yet to be integrated into macro models and concepts of the food environments. Such an undertaking is vital because the provision of food aid is rising in the UK and elsewhere [5] and the increasingly chronic nature of food poverty for vulnerable groups has made the issue a ‘public health emergency’ [6]. While food banks and other food aid outlets undeniably play a vital role in alleviating acute food insecurity, they are limited in their capacity to improve overall diet due to their provision of long life pre-packaged and processed foods for short-term alleviation of hunger [7]; in the United Kingdom most food banks limit clients to three visits each year, providing at most three days of food at each visit. If current food insecurity trends continue then the diets of low-income people may become characterised by the inclusion of significant amounts of donated and surplus food accessed via the third-sector. Developing ways of categorising and classifying food aid outlets and their services is therefore an important extension of health research that characterises and operationalises the contemporary food environment.

In order to fully understand contemporary dietary practices in the context of rising food poverty and an increasing reliance on food aid, a more holistic approach to characterising low-income food environments is needed. Such an undertaking is not without its challenges. The United Kingdom has yet to implement a national measure of household food insecurity like those used in Canada and the United States [8]. Thus far, attempts to pass a bill to that effect have yet to be successful. In which case, reliance on estimates of food insecurity based on factors such as the prevalence of food banks, the number of food parcels they distribute, and mapping populations at higher risk of food poverty as proxy measures is main the current approach [8].

Further challenges arise with regard to identifying food aid outlets. While the Trussell Trust, the United Kingdom’s largest food aid franchise, has data on its food banks and the number of food parcels they distribute in the public domain, efforts to create a directory of the varied non-Trussell Trust and independent food aid outlets in the United Kingdom are still ongoing (since 2017) [9]. Various third sector organisations have developed localised ‘food maps’ and subsidised food outlet directories. But these tend to be produced on an ad-hoc basis and not collated centrally by government (national or local). Progressive projects at the Local Authority level, mostly by Public Health teams, have been implemented to generate a more comprehensive picture of their local food environments – recording both retail and food aid outlets in recognition of the fact that residents on low incomes routinely use both (see [10] for a United Kingdom-based example of this).

Academic collaboration with local public health teams on such projects would be an appropriate way to expand the research agenda of food environments research while supporting the already overstretched local government teams. Working with local government and health teams to characterise neighbourhood food environments and measure food insecurity risk has been instrumental in developing methods and recommendations for national level approaches [8]. The logical next step is to combine these concerns and work collaboratively towards innovative ways of capturing and understanding local food environments (including food aid outlets) that can be used to inform food environments research more broadly. In addition, this work can inform provisioning of local food aid resources, as the responsibility of addressing household food insecurity is now held by these local government teams.

Food aid – in all its evolving forms – is embedded within both local food and welfare systems and is symptomatic of the broader trend of state retreat from the provision of welfare services. Concerns of food ‘access’, ‘choice’ and ‘affordability’ take on new meanings in this context, especially for this part of the ‘hidden’ food environment that requires membership fees, access via referral vouchers, pay-as-you-feel options, or very limited opening hours. Rethinking these concepts is necessary in order to generate knowledge of how the food aid and food banking environment influences the food practices of low income populations. Developing ways of capturing and understanding this expanded conceptualisation of the local food environment is necessary in order to both help build an evidence base to challenge policies that exacerbate the drivers of food insecurity and to inform interventions aimed at improving the diet of the most disadvantaged populations.
